# Supplementary material for: Gasdermin E is dispensable for H1N1 influenza virus pathogenesis in mice
Source: Microbiol Spectr. 2026 Feb 5;14(3):e02472-25. doi: 10.1128/spectrum.02472-25 (PMC12955486; doi:10.1128/spectrum.02472-25)
Supplement: Supplemental figures — Figures S1 and S2. [file spectrum.02472-25-s0001.docx]

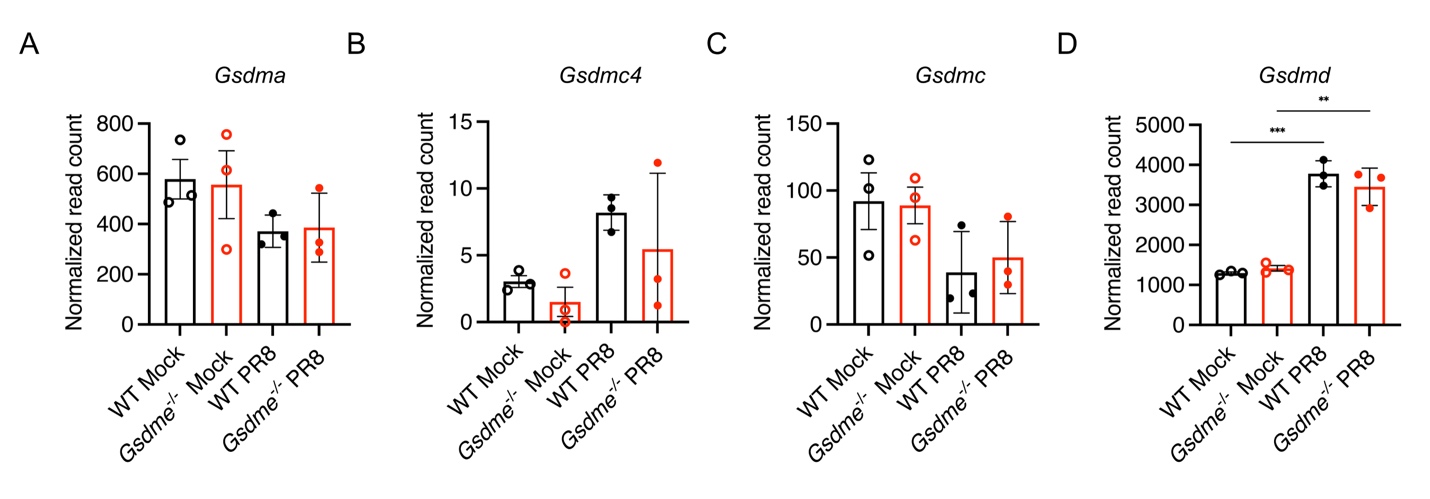


**Supplementary Figure 1: Gasdermin family member transcripts are not changed between WT and *Gsdme^-/-^* mice. A-D** DEseq2 normalized read counts for various gasdermin family genes.


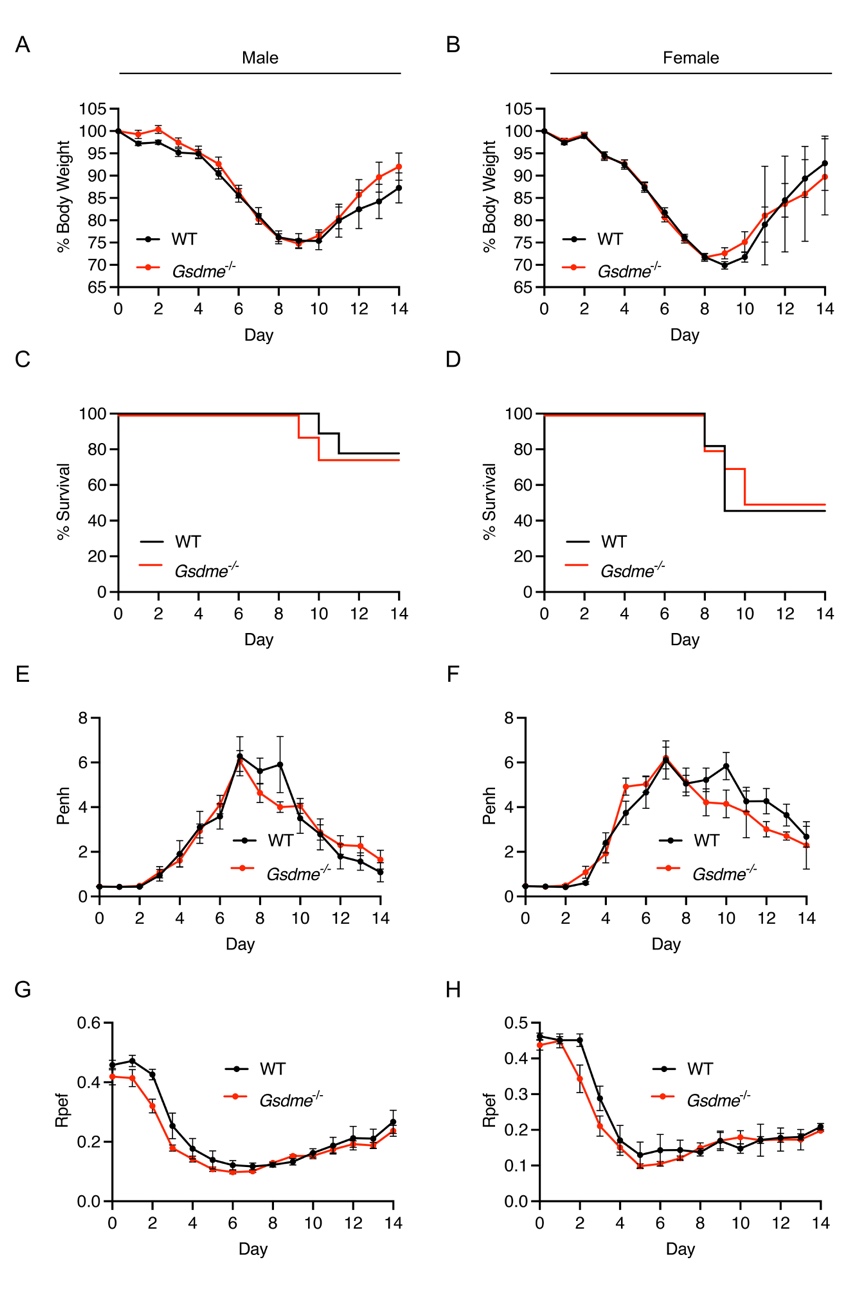


**Supplementary Figure 2: Sex disaggregation of data in Figure 4.** **A-H** Male and female WT and *Gsdme^-/-^* mice were intranasally infected with 100 TCID50 of minimally mouse adapted pandemic 2009 H1N1 IAV. **A-B** Weight loss measurements (**A** WT n = 17 and *Gsdme^-/-^* n = 15 for days 0-10, WT n = 7 and *Gsdme^-/-^* n = 8 for days 11-14, **B** WT n = 18 and *Gsdme^-/-^* n = 16 for days 0-10, WT n = 8 and *Gsdme^-/-^* n = 6 for days 11-14, **A-B** each dot is an average of individual mouse weights normalized to 100% relative to day 0, error bars indicate SEM, no significant differences between genotypes at any timepoint by two-way ANOVA followed by Bonferroni’s multiple comparisons test). **C-D** Survival analysis (**C** WT n = 9, *Gsdme^-/-^* n = 8, **D** WT n = 11 Gsdme^-/-^ n = 10, not significant by Log-rank Mantel-Cox test). **E-F** Enhanced pause (Penh) measurements from daily whole body plethysmography (**E** WT n = 7 and *Gsdme^-/-^* n = 6, **F** WT n = 8 and *Gsdme^-/-^* n = 6, each dot is an average of individual mouse Penh values for that day, error bars represent SEM, no significant differences between genotypes at any timepoint by two-way ANOVA followed by Bonferroni’s multiple comparisons test). **G-H** Ratio of time to peak expiratory flow (Rpef) measurements from daily whole-body plethysmography ((**E** WT n = 7 and *Gsdme^-/-^* n = 6, **F** WT n = 8 and *Gsdme^-/-^* n = 6, each dot is an average of individual mouse Rpef values for that day, error bars represent SEM, no significant differences between genotypes at any timepoint by two-way ANOVA followed by Bonferroni’s multiple comparisons test).
